# Epidemiological profile of leprosy in the state of Mato Grosso: descriptive study

**DOI:** 10.31744/einstein_journal/2021AO5622

**Published:** 2021-08-25

**Authors:** Aline Menezes Rossi Tavares

**Affiliations:** 1 Universidade Federal de Mato Grosso RondonópolisMT Brazil Universidade Federal de Mato Grosso, Rondonópolis, MT, Brazil.

**Keywords:** Leprosy, Communicable diseases, Epidemiology, descriptive, Physical therapy modalities, Rehabilitation

## Abstract

**Objective:**

To analyze the prevalence of leprosy cases using as parameters the number of diagnosed cases, age group, sex, clinical form of the disease, degree of physical disability, affected nerves, and therapeutic methods.

**Methods:**

This was a descriptive study. Data were collected in the Information System for Notifiable Diseases (*Sistema de Informações de Agravos de Notificação*) from 2014 to 2017 in the state of Mato Grosso (MT), Brazil.

**Results:**

In the studied period, 11,388 cases were notified in the state, with a higher prevalence in the year 2017. Most cases were diagnosed in individuals older than 15 years with a slight predominance of men. During diagnosis, there was prevalence of zero degree of physical disability and among individuals with affected nerves, mostly had less than five nerves affected.

**Conclusion:**

Analyzing the prevalence and epidemiological profile of leprosy cases in the state of Mato Grosso (MT) turns to be essential for coping with the disease, as it helps in its management and treatment, with an emphasis on multidisciplinary approach for the recovery of existing cases and prevention of new cases, especially at hyperendemic states.

## INTRODUCTION

Leprosy is a chronic infectious-contagious disease of slow evolution that is caused by *Mycobacterium Leprae*. This disease often affects skin and peripheral nerves specially in Schwann cells that is considered one of the main public health problems in Brazil. In addition, the commitment of peripheral neuropathy may contribute to permanent functional impairments, the disease still brings with itself one vicious circle of exclusion, stigma, and prejudice reflecting on social relations among these sick individuals.^(1,2)^

Brazil is highlighted as the second country in absolute number of leprosy cases. For some years, the State of Mato Grosso (MT), Brazil presents a hyperendemic level for leprosy, and the state occupy the first position in the country with the highest rates of prevalence and incidence of the disease. According to the Information System for Notifiable Diseases (Sinan) from 2010 to 2013, a total of 10,521 new cases were notified, and among transmissible disease leprosy is considered one of the main causes of permanent functional impairment.^(3-5)^

Leprosy, in addition to be characterized by appearance of spots on the skin, it may manifest changes on the face, upper and lower limbs. In relation to peripheral nervous system, the nerves often affected by the *Mycobacterium leprae* are: trigeminal, medium, ulnar, facial, auricle, radial, tibial, and common fibulae. The neural compromising that can occur even with the early diagnosis and adequate treatment of the disease lead to impairment and highly characteristic and peculiar deformity to each affected nerve, which can be reversal or not, depending on the observed degree.^(6,7)^

Although leprosy is considered a health problem, the disease has cure, and individuals affected by this affection might be treated, and conduct rehabilitation provided by the Brazilian Unified Health Unit (SUS) [*Sistema Único de Saúde*]. The goal of the treatment is cure, elimination of infection source and, consequently, to interrupt the transmission chain, being this strategy for the control of the disease.^(8)^ For patients with physical impairment, the rehabilitation recognizes the importance to address their needs, with the aim to include their activity in the family activities and their community, with equal citizenship, reducing all and any barrier for exclusion.^(2)^

Given that the health team is extremely crucial for the treatment and rehabilitation of sick individuals, it is paramount to use epidemiological data to improve planning of actions and perform treatment of diagnosed cases, in addition to adequate care plans for patients with sequelae of the disease.^(9)^

## OBJECTIVE

To analyze the prevalence of leprosy cases considering the number of diagnoses, age range, sex, clinical form of the disease, degree of physical impairment, affected nerves, and therapeutic methods.

## METHODS

This was a study based on secondary data obtained in the Sinan regarding the register of leprosy cases reported between 2014 and 2017 for the state of Mato Grosso, Center East region of Brazil. The State is organized in 22 microregions and five mesoregions, divided in 141 municipalities, which are the most populated and important regions, *i.e*., the capital Cuiabá, Várzea Grande, Rondonópolis, Sinop, Tangará da Serra, Barra do Garças and Cáceres, with estimative of 3,422 million of inhabitants.^(10)^

The studied period consisted from 2014 to 2017, and variables included were the number of diagnoses, age range, sex, clinical form of the disease, degree of physical impairment, affected nerves, and therapeutic methods.

The age range in this study corresponds to two categories: zero to 14 years and 15 years or older. For clinical form, we consider indeterminate, tuberculoid, dimorph, Virchowian, unclassified, and ignored. The physical inability was analyzed from the the diagnosis. The therapeutic methods described were paucibacillary polychemotherapy (PQT/PT, six doses), multibacillary polychemotherapy (PQT/MB, 12 doses), and another substitution method. To present results, we used tables and figures to show frequencies related to each collected information in the data system.

This study was conducted exclusively with publicly available secondary data without identification of subjects that followed ethical principles of the resolution 196/96 of the National Health Council, which justified the lack of approval from Ethical and Research Committee.^(11)^

## RESULTS

In the period considered for the study, a total of 11,388 cases of leprosy were notified. In 2014, according with Sinan, a total of 3 cases was observed with significant improvement in the following year for 3,639 cases, with prevalence of 11.14% per 10,000 people. In 2016, we registered 3,470 cases that corresponded to 10.49% per 10,000 people, being this last year with marked notifications.

The disease is commonly found among men (52.6%) with 15 years or older (94.6%) (Table 1).

**Table 1:** Cases of leprosy reported according to age range and sex

Age range (years)	Female n (%)	Male n (%)	Total n (%)
0-14	336 (2.9)	281 (2.5)	617 (5.4)
15 or more	5,067 (44.5)	5,704 (50.1)	10,771 (94.6)
Total	5,403 (47.4)	5,985 (52.6)	11,388 (100)

Source: Brasil. Ministério da Saúde. Tecnologia da Informação a Serviço do Sistema Único de Saúde (DATASUS). Hanseníase - Mato Grosso. Brasília (DF): Ministério da Saúde; 2008 [citado 2020 Mar 13]. Disponível em: http://tabnet.datasus.gov.br/cgi/dhdat.exe?hanseniase/hantfmt.def.^(12)^

**Figure 1:**
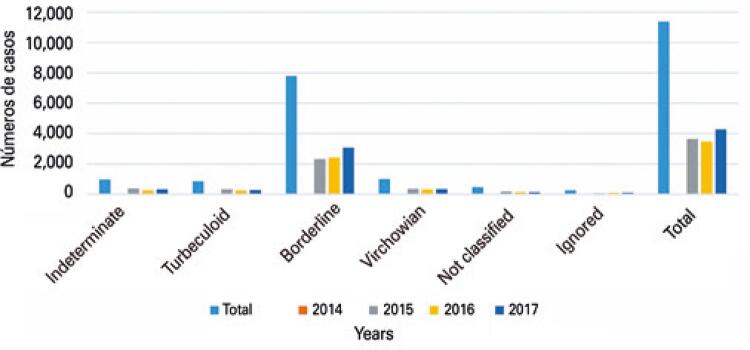
Number of leprosy cases reported among individuals in Mato Grosso according to clinical form of the disease

Source: Brasil. Ministério da Saúde. Tecnologia da Informação a Serviço do Sistema Único de Saúde (DATASUS). Hanseníase - Mato Grosso. Brasília (DF): Ministério da Saúde; 2008 [citado 2020 Mar 13]. Disponível em: http://tabnet.datasus.gov.br/cgi/dhdat.exe?hanseniase/hantfmt.def.^(12)^

Figure 1 relate the clinical format of the disease at the diagnosis. Of the total of diagnosed cases, the most common form of notification was dimorph (68.5%), with significant increase in year of 2016.^(12)^

Table 2 presents the physician impairment at the time of diagnosis, specifying the degree in each individual, and by sex. Most of individuals had degree 0, of these in 14.9% the impairment degree was not identified.^(12)^

**Table 2:** Leprosy cases in state of Mato Grosso with physical inability evaluated in diagnosis, according to sex

Variables	Degree of impairment					
	Degree 0 n (%)	Degree I n (%)	Degree II n (%)	Ignored/not evaluated n (%)	White n (%)	Total n (%)
Sex						
Male	2,781 (46.5)	1,816 (30.3)	377 (6.3)	734 (12.3)	277 (4.6)	5.985 (100)
Female	2,896 (53.6)	1,609 (29.8)	206 (3.8)	506 (9.3)	186 (3.5)	5.403 (100)

Source: Brasil. Ministério da Saúde. Tecnologia da Informação a Serviço do Sistema Único de Saúde (DATASUS). Hanseníase - Mato Grosso. Brasília (DF): Ministério da Saúde; 2008 [citado 2020 Mar 13]. Disponível em: http://tabnet.datasus.gov.br/cgi/dhdat.exe?hanseniase/hantfmt.def.^(12)^

Of 11,388 diagnosed individuals, 47% had five or less nerves affected, and the proportion of ignored cases was similar (47.5%). In relation to those that presented five or more affected nerves, by 2015 these cases corresponded to 1.3% and by 2017 cases increased to 2.3%.

**Figure 2:**
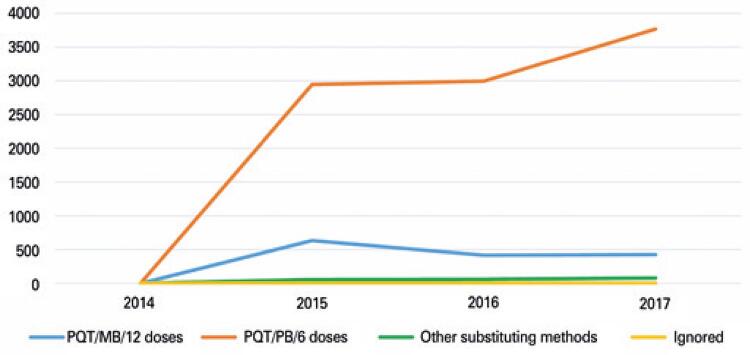
Therapeutic methods used by individuals diagnosed with leprosy

Source: Brasil. Ministério da Saúde. Tecnologia da Informação a Serviço do Sistema Único de Saúde (DATASUS). Hanseníase - Mato Grosso. Brasília (DF): Ministério da Saúde; 2008 [citado 2020 Mar 13]. Disponível em: http://tabnet.datasus.gov.br/cgi/dhdat.exe?hanseniase/hantfmt.def.^(12)^

In relation to therapeutic method used by those diagnosed with leprosy, the PQT/MB/12 doses were showed in 85.2% of them. Its use increased in function of time, and there is reduction of PQT/PB/6 doses: in 2015, corresponded to 5.5% of cases, and in the following year, it decreased by 3.6%. Still, we highlight that other therapeutic methods corresponded only to 1.7% (Figure 2).

PQT/PB/6 doses: paucibacillary polychemotherapy, six doses; PQT/MB/12 doses: multibacillary polychemotherapy, 12 doses.

## DISCUSSION

The endemic situation of the disease is supported in terms of morbidity, persistent transmission, and late diagnosis, facts that broads the relevance of leprosy as a public health issue.^(13)^

In this study, the year with highest number of case reports was 2017, and for the chosen time series the majority of patients with leprosy were men aged 15 years or older, with clinical dimorph and 0 degree of physical impairment. Of the sample, 47% had affected five or less nerves, and the most evidence therapeutic method was PQT/MB/12 doses (85.2%).

In relation to age range, the state of Mato Grosso had the highest index of the disease in individuals aged 15 years or older, equivalent to 94.6%. In agreement with this study, in the state of Espírito Santo from 1988 to 1995 the tendency of increasing occurred within an age range from 15 to 19 years, 20 to 29 years, and 50 years or older, a equivalent rate for 6% yearly.^(14)^ In relation to prevalence of the disease among children, studies have highlighted that, from 2003, Mato Grosso, along with Bahia, Tocantins, Rondônia, Pará and Maranhão had a significant increase of cases among patients younger than 15 years.^(13)^

The percentage of reports in men was 52.6%. Moreira et al.,^(14)^ also checked the finding that corroborate to other studies showing little predominance among men. Studies have related that, in terms of percentual distribution per sex, there is discrete predominance of affection among men with a mean of 1.1 for man and 1 for woman.

According to epidemiological bulletin in 2018 among men the rate was high, reaching 15,17 cases per 1 million while in women the rate was 6,07 cases per 1 million. These data are relevant, in addition to point out that leprosy is a relatively higher problem among men, the population group with the lowest frequency in health units.^(4)^

There is still a predominance of dimorph clinical form with 68.5% and significant increase by 2016, a fact that was also evidence in a study conducted by Lima et al.,^(15)^ in which dimorph form corresponded to 58.5% of cases followed by Virchowian form with rate of 19.6% of cases.

In one study on physical impairment among individuals affected by leprosy in the period after discharge of polychemotherapy in the municipality of North region of Brazil, predominant to indeterminate clinical forms and dimorph, showing a higher percentage of paucibacillary cases and follow by national and state epidemiologic clinical standards.^(16)^

In this study, most of individuals had 0 degree of impairment, and 14.9% did not identify the impairment degree. Studies pointed that, in relation to detection of new cases with degree 2 of physical impairment, *i.e*., in which deformities on hands, feet or eyes are visible, the studies have showed a mean rate of 10.53 cases for each 1 million of inhabitant in Brazil, from 2012 to 2016.^(4)^

Regarding variable affected of nerves, of the 11,388 diagnosed individuals 47% had five to less nerves affected, and the proportion of ignored cases was similar (47.5%). This variable, although relevant, indicated the fragility in which reliable data was obtained in Sinan database.

In relation to therapeutic method used by individuals diagnosed with leprosy, the PQT/MB/12 doses were mentioned by 85.2% of them. Definition of therapeutic method depends on the operational classification of case, using rifampicin’s bactericidal, dapsone’s bacteriostatic, and clofazimine effects. For paucibacillary case, the discharge after cure can be obtained after six doses monthly. To the multibacillary cases, the method included 12 doses monthly.^(6)^

In a study conducted by Machado et al., in the municipality of Alta Floresta (MT), among the participants 86.54% were classified as multibacillary and only 13.56% as paucibacillary, therefore, resulting in the form of the use of this multibacillary method for treatment of most individuals, a fact that was similar in the present study. Authors considered the predominance of multibacillary cases revealing areas of intense dissemination of leprosy and also suggested late diagnosis resulting in the increase of the incidence of physical impairment among sick individuals and also suggested failures in examination.^(17)^

The present study has limitations. It is important to highlight that, because this disease reporting is compulsory and its information were extracted from a database, data can present failures, which may compromise the reliability of this study findings. The under reporting major weakness of this study, because this was strongly evident in the year of 2014 that only three individuals was reported in the state of Mato Grosso.

According to the World Health Organization (WHO) only in 2016 the Global Leprosy Strategy 2016-2020 was built with the goal to accelerate actions for leprosy free world including the National Program of Leprosy Control that aimed to guide the practice in services at all levels and of different complexities, according to the SUS, strengthening the epidemiological surveillance of leprosy.^(18)^

Other limitation is concerned the inability to conduct some calculations to obtain rates of prevalence for some variables, due to the lack of updated data from the Brazilian Institute of Geography and Statistics (IBGE).

## CONCLUSION

Among the studies years, 2017 was the year that presented the highest prevalence of the disease that can be explained by the increase of endemicity in the state or the advance/use of the system of compulsory report. A system that, in 2014, presented weakness such as under reporting, resulting in the report of three cases only occurring in the entire state of Mato Grosso.

Of individuals reported with leprosy between 2014 to 2017, most of them had 15 years or more, and it showed a discrete predominance in men. Dimorph clinical form was the most evident one. Most individuals had 0 degree of physical impairment, affecting five peripheral nerves or less, and with the use of multibacillary therapeutic method.

Analyzing the prevalence and epidemiological profile of leprosy cases in the state of Mato Grosso turns to be essential for coping with the disease, as it helps in its management and treatment, with an emphasis on multidisciplinary approach for the recovery of existing cases and prevention of new cases, especially at hyperendemic states.
