# Supplementary material for: Epidemiological profile of leprosy in the state of Mato Grosso: descriptive study
Source: Einstein (Sao Paulo). 2021 Aug 25;19:eAO5622. doi: 10.31744/einstein_journal/2021AO5622 (PMC8386686; doi:10.31744/einstein_journal/2021AO5622)
Supplement: Translation

# Translation: Perfil epidemiológico da hanseníase no estado de Mato Grosso: estudo descritivo

**Authors:** Aline Menezes Rossi Tavares

**Affiliations:** 1 Brasil Universidade Federal de Mato Grosso, Rondonópolis, MT, Brasil.

**Keywords:** Hanseníase, Doenças transmissíveis, Epidemiologia descritiva, Modalidades de fisioterapia, Reabilitação

## Abstract

**Objetivo:**

Analisar a prevalência de casos de hanseníase, tendo como parâmetros número de casos diagnosticados, faixa etária, sexo, forma clínica da doença, grau de incapacidade física, nervos acometidos e esquema terapêutico.

**Métodos:**

Trata-se de estudo descritivo. Os dados foram coletados no Sistema de Informações de Agravos de Notificação, referentes ao período de 2014 a 2017 e relativos ao estado do Mato Grosso (MT), Brasil.

**Resultados:**

No período em estudo notificaram-se 11.388 casos no estado, com maior prevalência no ano de 2017. A maior parte dos casos diagnosticados foi em indivíduos com mais de 15 anos, com discreto predomínio no sexo masculino. Prevaleceu o grau zero de incapacidade física durante o diagnóstico e menos de cinco nervos em cada indivíduo, naqueles com nervos afetado.

**Conclusão:**

A análise da prevalência e do perfil epidemiológico de casos da hanseníase no estado de Mato Grosso (MT) torna-se essencial para o enfrentamento da doença, pois auxilia no manejo e no tratamento, com ênfase no trabalho multiprofissional para recuperação dos casos já existentes e na prevenção de novos casos, especialmente em estados hiperendêmicos.

## INTRODUÇÃO

A hanseníase, uma doença crônica, infectocontagiosa, de evolução lenta, cujo agente etiológico é *Mycobacterium leprae*, que tem predileção em acometer pele e nervos periféricos, em especial as células de Schwann, é considerada um dos principais problemas de saúde pública no Brasil. Além de provocar o comprometimento neurológico periférico, que pode contribuir para incapacidades funcionais permanentes, a doença traz ainda consigo um ciclo vicioso de exclusão, estigma e preconceito, refletido nas relações sociais desses doentes.^(1,2)^

O Brasil destaca-se como o segundo país em número de casos absolutos. Há alguns anos, o estado do Mato Grosso (MT), Brasil apresenta nível hiperendêmico para casos de hanseníase, ocupando a primeira posição, com as maiores taxas de prevalência e incidência da doença no país. De acordo com o Sistema de Informação de Agravos de Notificação (Sinan), de 2010 a 2013, foram notificados 10.521 casos novos, sendo que, entre as doenças transmissíveis, a hanseníase é considerada uma das principais causas de incapacidade física permanente.^(3-5)^

A hanseníase, além de ser caracterizada pelo surgimento de manchas na pele, pode evoluir ainda para alterações na face e nos membros superiores e inferiores. Em relação ao sistema nervoso periférico, os nervos frequentemente acometidos por *Mycobacterium leprae* são: trigêmeo, mediano, ulnar, facial, auricular, radial, tibial e fíbular comum. O comprometimento neural, que pode ocorrer até mesmo com o diagnóstico precoce e tratamento adequado da doença, ocasiona incapacidades e deformidades muito características e peculiares a cada um dos nervos afetados, podendo ser reversíveis ou não, de acordo com o grau observado.^(6,7)^

Embora seja considerado um problema de saúde pública, a hanseníase tem cura, e os indivíduos acometidos dispõem de tratamento e reabilitação ofertados pelo Sistema Único de Saúde (SUS). O tratamento objetiva a cura, a eliminação da fonte de infecção e, dessa forma, o interrompimento da cadeia de transmissão, sendo uma estratégia para o controle da doença.^(8)^ Para pacientes com incapacidades físicas, a reabilitação reconhece a importância de atender suas necessidades, com o intuito de incluí-lo ativamente nas atividades familiares e de sua comunidade, com igualdade de cidadania, eliminando toda e qualquer barreira de exclusão.^(2)^

Tendo em vista que a equipe de saúde apresenta extrema importância para o tratamento e a reabilitação dos doentes, torna-se imprescindível a utilização de dados epidemiológicos, para melhor planejamento das ações e da condução do tratamento dos casos diagnosticados, além de adequar planos de cuidado para os pacientes com sequelas da doença.^(9)^

## OBJETIVO

Analisar a prevalência de casos de hanseníase tendo como parâmetros número de casos diagnosticados, faixa etária, sexo, forma clínica da doença, grau de incapacidade física, nervos acometidos e esquema terapêutico.

## MÉTODOS

Trata-se de estudo sobre dados secundários, obtidos do Sinan, referentes ao registro de casos de hanseníase notificados entre os anos de 2014 e 2017 para o estado de Mato Grosso (MT), localizado na região Centro-Oeste do Brasil. O estado está organizado em 22 microrregiões e cinco mesorregiões, dividindo-se em 141 municípios, sendo os mais populosos e importantes a capital Cuiabá, Várzea Grande, Rondonópolis, Sinop, Tangará da Serra, Barra do Garças e Cáceres, com estimativa de 3,442 milhões de habitantes.^(10)^

O período selecionado correspondeu aos anos de 2014 a 2017, tendo como variáveis o número de casos diagnosticados, a faixa etária, o sexo, a forma clínica da doença, o grau de incapacidade física, os nervos acometidos e o esquema terapêutico.

A faixa etária operacionalizada no presente estudo corresponde a duas categorias: zero a 14 anos e 15 anos e mais. Para forma clínica, consideraram-se indeterminada, tuberculoide, dimorfa, virchowiana, não classificada e ignorada. A incapacidade física foi analisada a partir do diagnóstico. Os esquemas terapêuticos descritos foram poliquimioterapia paucibacilar (PQT/PB, seis doses), poliquimioterapia multibacilar (PQT/MB, 12 doses) e outros esquemas substitutivos. Para a apresentação dos resultados, utilizaram-se tabelas e figuras, que expõem as frequências relativas de cada informação coletada no sistema de dados.

O presente estudo foi realizado exclusivamente com dados secundários, de acesso público, sem identificação dos sujeitos, obedecendo aos princípios éticos da resolução 196/96 do Conselho Nacional de Saúde, o que justifica a ausência do parecer do Comitê de Ética em Pesquisa.^(11)^

## RESULTADOS

No período considerado para o estudo, foram notificados 11.388 casos de hanseníase Em 2014, de acordo com o Sinan, observaram-se apenas três casos, com aumento significativo no ano seguinte para 3.639 casos, com prevalência de 11,14% por 10 mil pessoas; em 2016, registraram-se 3.470 casos, que corresponderam a 10,49% por 10 mil pessoas e, em 2017, foram 4.276 casos e prevalência de 12,78% por 10 mil pessoas – sendo este o último ano com notificações divulgadas.

A doença predominou em indivíduos com 15 anos ou mais (94,6%) e no sexo masculino 52,6% (Tabela 1).

**Tabela 1:** Casos de hanseníase notificados de acordo com faixa etária e sexo

Faixa etária (anos)	Feminino n (%)	Masculino n (%)	Total n (%)
0-14	336 (2,9)	281 (2,5)	617 (5,4)
15 ou mais	5.067 (44,5)	5.704 (50,1)	10.771 (94,6)
Total	5.403 (47,4)	5.985 (52,6)	11.388 (100)

Fonte: Brasil. Ministério da Saúde. Tecnologia da Informação a Serviço do Sistema Único de Saúde (DATASUS). Hanseníase - Mato Grosso. Brasília (DF): Ministério da Saúde; 2008 [citado 2020 Mar 13]. Disponível em: http://tabnet.datasus.gov.br/cgi/dhdat.exe?hanseniase/hantfmt.def.^(12)^

A figura 1 relaciona a forma clínica da doença no momento de seu diagnóstico. Do total de casos diagnosticados, a forma mais notificada foi a dimorfa (68,5%), com aumento significativo no ano de 2016.^(12)^

**Figura 1:** Número de casos de hanseníase de indivíduos notificados no Mato Grosso, segundo a forma clínica da doença

Fonte: Brasil. Ministério da Saúde. Tecnologia da Informação a Serviço do Sistema Único de Saúde (DATASUS). Hanseníase - Mato Grosso. Brasília (DF):Ministério da Saúde; 2008 [citado 2020 Mar 13]. Disponível em: http://tabnet.datasus.gov.br/cgi/dhdat.exe?hanseniase/hantfmt.def.^(12)^

A tabela 2 apresenta o grau de incapacidade física no momento do diagnóstico, especificando o grau em cada indivíduo e por sexo. A maioria dos indivíduos apresentou grau 0, sendo que, em 14,9% não foi identificado o grau de incapacidade.^(12)^

**Tabela 2:** Casos de hanseníase no estado de Mato Grosso com grau de incapacidade física avaliado no diagnóstico, segundo sexo

Variáveis	Grau de incapacidade					
	Grau 0 n (%)	Grau I n (%)	Grau II n (%)	Ignorado/não avaliado n (%)	Branco n (%)	Total n (%)
Sexo						
Masculino	2.781 (46,5)	1.816 (30,3)	377 (6,3)	734 (12,3)	277 (4,6)	5.985 (100)
Feminino	2.896 (53,6)	1.609 (29,8)	206 (3,8)	506 (9,3)	186 (3,5)	5.403 (100)

Fonte: Brasil. Ministério da Saúde. Tecnologia da Informação a Serviço do Sistema Único de Saúde (DATASUS). Hanseníase - Mato Grosso. Brasília (DF): Ministério da Saúde; 2008 [citado 2020 Mar 13]. Disponível em: http://tabnet.datasus.gov.br/cgi/dhdat.exe?hanseniase/hantfmt.def.^(12)^

No que se refere ao acometimento de nervos, dos 11.388 indivíduos diagnosticados, 47% apresentaram acometimento de cinco ou menos nervos, e a proporção de casos ignorados foi similar (47,5%). Em relação aos que apresentaram cinco ou mais nervos acometidos, em 2015, esses casos corresponderam a 1,3% e, em 2017, aumentaram para 2,3%.

Em relação ao esquema terapêutico utilizado pelos indivíduos diagnosticados com hanseníase, a PQT/MB/12 doses foi evidenciada em 85,2% deles. Seu uso aumentou em função do tempo, e houve a redução da PQT/PB/6 doses: em 2015, correspondeu a 5,5% dos casos e, no ano seguinte, reduziu a 3,6%. Destaca-se ainda que outros esquemas terapêuticos corresponderam a apenas 1,7% (Figura 2).

**Figura 2:** Esquema terapêutico utilizado pelos indivíduos diagnosticados com hanseníase

Fonte: Brasil. Ministério da Saúde. Tecnologia da Informação a Serviço do Sistema Único de Saúde (DATASUS). Hanseníase - Mato Grosso. Brasília (DF):Ministério da Saúde; 2008 [citado 2020 Mar 13]. Disponível em: http://tabnet.datasus.gov.br/cgi/dhdat.exe?hanseniase/hantfmt.def.^(12)^

PQT/PB/6 doses: poliquimioterapia paucibacilar, seis doses; PQT/MB/12 doses: poliquimioterapia multibacilar, 12 doses.

## DISCUSSÃO

A endemia da doença encontra-se sustentada na força de sua morbidade, na transmissão persistente e no diagnóstico tardio, fato que amplia a relevância da hanseníase como questão de saúde pública.^(13)^

Neste estudo, o ano com maior número de casos notificados foi 2017, sendo que, para a série temporal escolhida, os pacientes com hanseníase, em sua maioria, eram da faixa etária de 15 anos ou mais, homens, com a forma clínica dimorfa e grau 0 de incapacidade física. Apresentaram acometimento de cinco ou menos nervos 47% da amostra, e o esquema terapêutico mais evidenciado foi PQT/MB/12 doses (85,2%).

Ao que se refere à faixa etária, o estado do Mato Grosso apresenta maior índice da doença em indivíduos com 15 anos ou mais, equivalente a 94,6%. Em consonância com este estudo, no estado do Espírito Santo, no período de 1988 a 1995, a tendência crescente ocorreu para as faixas etárias de 15 a 19 anos, 20 a 29 anos e 50 anos e mais - equivalente a 6% ao ano.^(14)^ No que se refere à prevalência da doença em crianças, alguns estudos destacam que, a partir de 2003, o Mato Grosso, juntamente de Bahia, Tocantins, Rondônia, Pará e Maranhão, apresentou aumento significativo de casos em menores de 15 anos.^(13)^

O percentual de notificações em indivíduos do sexo masculino foi de 52,6%. Moreira et al.,^(14)^ também verificaram achados que corroboram os de outras pesquisas, evidenciando discreto predomínio no sexo masculino. Pesquisas revelam que, na distribuição percentual por sexo, há predomínio discreto de acometimento do sexo masculino, com média de 1,1 homem para uma mulher.

Segundo o boletim epidemiológico divulgado em 2018, no que se refere ao sexo masculino, a taxa foi bem mais elevada, atingindo 15,17 casos por 1 milhão de homens, enquanto, no sexo feminino, a taxa foi de 6,07 casos para cada 1 milhão de mulheres. Esses dados são relevantes, além de apontar que a hanseníase é um problema relativamente maior entre os homens, grupo populacional que tem menor frequência nas unidades de saúde.^(4)^

Houve predominância ainda da forma clínica dimorfa com 68,5% e aumento significativo no ano de 2016, fato que também foi evidenciado na pesquisa realizada por Lima et al.,^(15)^ em que a forma dimorfa correspondeu a 58,5% dos casos, seguido pela forma virchowiana com 19,6%.

Em um estudo sobre incapacidade física em pessoas acometidas pela hanseníase no período pós-alta da poliquimioterapia em um município da região Norte do Brasil, predominaram as formas clínicas indeterminadas e dimorfa, havendo maior percentual dos casos paucibacilares e acompanhando o padrão clínico epidemiológico nacional e estadual.^(16)^

Neste estudo, a maioria dos indivíduos apresentou grau de incapacidade 0, sendo que, em 14,9%, não identificou-se o grau de incapacidade. Pesquisas apontam que, em relação à detecção de casos novos com grau 2 de incapacidade física, ou seja, no qual já são visíveis deformidades nas mãos, nos pés ou nos olhos, os estudos revelaram taxa média de 10,53 casos para cada 1 milhão de habitantes do Brasil, no período de 2012 a 2016.^(4)^

Em relação à variável acometimento de nervos, dos 11.388 indivíduos diagnosticados, 47% apresentaram acometimento de cinco ou menos nervos, e a proporção de casos ignorados foi similar (47,5%). Essa variável, embora relevante, aponta para a fragilidade em se obterem dados fidedignos na base de dados Sinan.

Em relação ao esquema terapêutico utilizado pelos indivíduos diagnosticados com hanseníase, a PQT/MB/12 doses foi evidenciada em 85,2% deles. A definição do esquema terapêutico depende da classificação operacional do caso, utilizando-se o bactericida rifampicina e os bacteriostáticos dapsona e clofazimina. Para os casos paucibacilares, a alta por cura pode ser obtida após seis doses mensais. Já para os casos multibacilares, o esquema compreende 12 doses mensais.^(6)^

Em estudo realizado por Machado et al., no município de Alta Floresta (MT), dentre os participantes, 86,54% foram classificados como multibacilares e apenas 13,56% como paucibacilares, resultando, dessa forma, no uso do esquema multibacilar para tratar a maioria dos indivíduos, fato que se assemelha ao presente estudo. Os autores consideram que a predominância de casos multibacilares revela áreas de intensa disseminação da hanseníase e também sugere diagnóstico tardio, resultando no aumento da incidência de incapacidades físicas nos doentes e sugerindo também falhas na realização do exame.^(17)^

O presente estudo apresenta limitações. É importante ressaltar que, sendo esta uma doença de notificação compulsória e cujas informações foram extraídas de uma base de dados, elas podem conter falhas, o que compromete a fidedignidade dos achados apresentados. A subnotificação é uma grande fragilidade deste estudo, pois foi fortemente evidenciada no ano de 2014, em que foram notificados no estado de Mato Grosso apenas três indivíduos.

Segundo a Organização Mundial da Saúde (OMS), apenas em 2016 foi elaborada a Estratégia Global para Hanseníase 2016-2020, que objetivou acelerar a ação rumo a um mundo sem a doença, as quais englobam o Programa Nacional de Controle da Hanseníase (PNCH), que visa orientar a prática em serviços em todas a instâncias e diferentes complexidades, de acordo com o SUS, fortalecendo as ações de vigilância epidemiológica da hanseníase.^(18)^

Outra fragilidade é em relação à impossibilidade de realizar alguns cálculos para obtenção de taxas de prevalência para algumas variáveis, devido à inexistência de dados atualizados provenientes do Instituto Brasileiro de Geografia e Estatística (IBGE).

## CONCLUSÃO

Dentre os anos estudados, o de 2017 foi o que apresentou maior prevalência para a doença, o que pode ser explicado pelo aumento da endemicidade no estado ou avanço/uso do sistema de notificação compulsória, sistema este que, em 2014, apresentou fragilidades, como a subnotificação, resultando na notificação de apenas três casos em todo o estado de Mato Grosso.

Dos indivíduos notificados com hanseníase entre 2014 a 2017, maioria possuía 15 anos ou mais, e foi discreta a predominância do sexo masculino. A forma clínica mais evidenciada foi a dimorfa. A maioria dos indivíduos possuía grau 0 de incapacidade física, com acometimento de cinco nervos periféricos ou menos e em uso do esquema terapêutico multibacilar.

A análise da prevalência de casos da hanseníase no estado de Mato Grosso torna-se essencial para o enfrentamento da doença, visto que auxilia no manejo e no tratamento, com ênfase no trabalho multiprofissional para recuperação dos casos já existentes e prevenção de novos casos, especialmente em estados hiperendêmicos.
